# Impact of work demand constraints on psychological distress through workplace bullying and personality traits: A moderated-mediation model

**DOI:** 10.3389/fpsyg.2022.965835

**Published:** 2023-03-02

**Authors:** Khalida Naseem, Majid Ali

**Affiliations:** ^1^Faculty of Economics and Management Sciences, School of Business and Management, Minhaj University Lahore, Lahore, Pakistan; ^2^Department of Economics and Agri Economics PMAS-UAAR, University Rawalpindi, Rawalpindi, Pakistan

**Keywords:** workplace bullying, work demand constraints, psychological distress, personality traits, counseling, M12, O15

## Abstract

**Introduction:**

This study addressed the human aspects of sustainable development in organizations by applying work demand resource theory and the main focus of the study is to investigate the negative aspects of workplace bullying on human’s mental health. This study investigated how the work demand constraints play a role in increasing psychological distress among employees through the mediation of workplace bullying. This study also considers personality traits that play a role in preventing psychological distress resulting from workplace bullying.

**Methods:**

The authors collected data by means of a self-administered questionnaire. The questionnaire was distributed among 1000 employees selected using a systematic sampling technique, patronized among three service sectors: the health sector, education sector, and forest department in the city of Lahore in Pakistan. The data was analyzed by employing Partial Least Square Structural Equation Modeling (PLS-SEM) using Smart PLS 3.3.3.

**Results:**

Results of this study reveal that work demand constraints (WDC) play a significant role in workplace bullying and impact increasing psychological distress. Personality traits play a moderating role between work demand constraints and workplace bullying behavior on the one hand and psychological distress on the other hand; however, openness to experiences was found to have a moderating relationship between work demand constraints and workplace bullying. Meanwhile, agreeableness and openness to experiences were found to have a moderating relationship between work demand constraints (WDC) and psychological distress.

**Discussion:**

This study also has practical implications for employers, such as providing psychological counseling, personality development training at the workplace etc. The relationship of work demand constraints with psychological distress and workplace bullying through direct and indirect moderating effects of personality traits in Pakistan’s service sector are unique contributions of this study.

## Introduction

Organizational sustainability has received a great deal of attention from academia and business realms for the last few years (de Freitas et al., 2017; Lopes et al., 2017; Yu et al., 2018; Contreras and Abid, 2022; Elahi et al., 2022). Out of the triple bottom-line dimensions of organizational sustainability, unfortunately, the social dimension associated with human sustainability is not dealt with by academia in a balanced way in contrast to environmental and economic dimensions (Ahmad and Thaheem, 2017; Eizenberg and Jabareen, 2017; Abid et al., 2020; Ilyas et al., 2020). The social dimension of sustainable organizations is the human dimension related to employees’ well-being (Di Fabio, 2017). This demonstrates that greater emphasis should be placed on the social dimension, which is the research gap. This study makes an effort to bridge this gap in the literature. Human sustainability has been defined by Pfeffer (2010) as management practices that have a profound influence on the employees’ physical and psychological well-being sustainably. Literature suggests that sustainable organizations achieve this dimension by enhancing employee wellbeing, i.e., by increasing positive aspects and reducing negative aspects of human development (Ashfaq et al., 2021; Khan et al., 2021; Qaiser and Abid, 2022). Likewise, a few researchers mentioned that thriving organizations could make their employees motivated and blissful (Abid and Contreras, 2022; Abid et al., 2022). There is a diverse array of factors that affect employee well-being to improve workplace outcomes for sustainable organizations (Chughtai et al., 2015; Ilyas et al., 2022). Few factors have a positive impact, and others have a negative impact on employee well-being.

Bullying at the workplace is a force that has a negative impact on human sustainability by engendering psychological distress among employees. Its presence is not only incompatible with a healthy and sustainable work environment but also has a psychological, and social consequences for individuals (Gómez-Galán et al., 2021). Due to its negative association with psychological well-being; literature requires its further investigation and exploration in different work environments (Peña-Casares and Aguaded-Ramírez, 2021; Carretero Bermejo et al., 2022). There is great interaction between bullying and human sustainability and this topic still requires further investigation from different perspectives (Cullinan et al., 2020; Conway et al., 2021). Based on this gap this study investigated the impact of bullying on psychological distress. Bullying is defined as “repeatedly harassing, offending, socially excluding or targeting someone at work with negative acts for a prolonged period” (Leymann, 1996; Baillien et al., 2017). It is associated with incapability to concentrate, mood swings, sleep problems, anxiety, fear, and depressive symptoms (Verkuil et al., 2015; Karatza et al., 2016; O’Donnell and MacIntosh, 2016; Agostini et al., 2019). It is also associated with psychological and psychosomatic symptoms such as headaches, respiratory, and cardiac complaints, hypertension and hypersensitivity to sounds (Hallberg and Strandmark, 2006; Devonish, 2017; Peña-Casares and Aguaded-Ramírez, 2021).

Evidence suggests that almost 7% of employees experienced bullying in Jordan, 20.3% experienced bullying in Basque, and 13.3% experienced bullying in Taiwan (Nakayama, 2019; Shahrour et al., 2020). The hidden costs of bullying behaviors were examined in a study and found that €5323.01 was reported for medical treatments and also indirect costs in the form of productivity loss (Jantzer et al., 2019). Another study found that the annual estimated cost for productivity loss was reported as €51.8 million in the public sector, and €187.6 million in the private sector (Cullinan et al., 2020).

Researchers (Bakker and Demerouti, 2017; Finstad et al., 2019; Balducci et al., 2020) indicated workplace bullying is a consequence of the job demand constraints. Research also found that a stressful workplace environment often leads to worsened interpersonal relationships, thus leading to workplace bullying (Zhao et al., 2020). Due to the experience of workplace bullying, there is a growing tendency that the target may suffer deteriorated health issues such as physical, mental, emotional, or psychological illness (Bryson et al., 2020). Prime reason for these negative behaviors is inherent in stress because of work demands (Zahlquist et al., 2019). Therefore, this study uses job demand-resource theory to investigate the effects of work demand constraints on bullying and psychological distress.

Based on the above literature, we assume that workplace bullying is a major issue, but the question is still unclear whether workplace bullying can mediate the effect of work-related stress on employees’ physical, mental, or psychological health (Finstad et al., 2019). Therefore, the first objective of this study is to investigate the impact of work-related stressors such as work demand constraints on psychological distress through the mediating role of workplace bullying. The second objective of this study is to investigate the moderating role of personality traits (Extroversion, openness to experience, neuroticism, conscientiousness, and agreeableness). We used two waive time lagged data, quantitatively estimated the mediation effect, and tested its significance.

To reach our objectives, this study puts the following questions.

1) Does WDC positively influence workplace bullying?
2) Does workplace bullying mediate the relationship between WDC and psychological distress?
3) Does Personality traits (extroversion, agreeableness, openness to experience, neuroticism, and conscientiousness) moderate the positive relationship between WDC and bullying behavior?
4) Does Personality traits (extroversion, agreeableness, openness to experience, neuroticism, and conscientiousness) moderate the indirect impact of WDC on psychological distress through bullying behavior?

The paper is organized as follows. Section “Literature review and hypotheses development” entails hypotheses formulation after reviewing the pertinent literature. Section “Method” covers methodology and measures of the current study, in section “Results” results of the study are presented. Section “Discussion” describes the discussion and finally, implications, limitations, future directions, and conclusion are discussed.

## Literature review and hypotheses development

### Work demand constraints as an antecedent of workplace bullying

In research on workplace bullying, Samsudin et al. (2020) highlighted the necessity of organizational antecedents of bullying and considered these a main cause of bullying (Baillien et al., 2019). On the other hand, Zapf and Einarsen (2020) investigated that individual elements were the responsible for workplace bullying. Therefore, there is room to search for either reason for bullying. Consequently, bullying must be taken as a dyadic interaction between individuals, where neither personal nor situational factors are enough to describe its reason of existence. Organization and its management also play an intervening role between bullying and conflict, so it is concluded that a wide range of factors such as individual, situational, organizational, dyadic, group, contextual and societal level factors may each be the critical cause of bullying (Hoel et al., 2001; Gómez-Galán et al., 2021). This study considers “organizational” factors, either providing support to either model or not.

Organizational factors influencing the frequency of bullying, among others, are chaotic and unpredictable work environment, reduced work control, lack of procedural justice, destructive management style, and ethical climate (Ahmed and Omran, 2020; Samsudin et al., 2020). However, there is a scarcity of empirical research, and it is not clear yet which factors in the workplace environment increase bullying or under which mechanism a poor workplace breeds bullying (Samsudin et al., 2020). Research focused on work related stressors as antecedents of workplace bullying, such as job design, management practices, and social context (Feijó et al., 2019). This study considers a work-related antecedent, work demand constraints as an antecedent of workplace bullying.

### Work demand-resource theory

Research on employee well-being has been guided by job demands-Resource theory put forward by Demerouti (Demerouti et al., 2001; Bakker and Demerouti, 2017). The main idea of job demands-resource theory is based on two job characteristics such as job demands and job resources. Job demands are the physical and emotional stressors in individual’s role such as time pressures, workload, stressful environment, emotional labor (Bakker and Demerouti, 2017). While job resources are those aspects of the job that are required in obtaining work objectives and motivates for professional growth and development such as autonomy, strong work relationships, opportunities for advancement, and learning and development (Schaufeli and Bakker, 2004). An extension in job demands resource theory was conducted in the form of personal resources (Bakker and Demerouti, 2007) which is based on belief system of the humans about how much they have control on environment. The theory job demands-resource theory proposes that high job demands and job resources and personal resources activate multiple processes at work (Demerouti et al., 2001). Job demands is associated with psychological and mental health damage processes: having high job demands or demand constraints like, workload, task overburdening, and inadequate infrastructure leads to overthinking and in the end psychological disorders (Medzo-M’engone, 2021). In contrast, job resources clues to enhanced motivational process: with more job resources employees moves to more job engagement and becomes more dedicated toward work (Bakker and Demerouti, 2017). Job demands and resources also interrelate to forecast strain (Dicke et al., 2018). For example, in the presence of high autonomy (job resources), a person may deal with high work burden (high job demand). Generally, work demand constraints, where excessive workloads and overburdening with limited authority and resources consume more individual resources and make them stressed (Naseem and Ahmed, 2020). This shows that high job demands interacting with negative behaviors leads to more emotionally exhaustion.

In service sector, specifically health care units and academia, resources are related to funding possibilities, administrative staff, managerial skills, and access to higher studies. Resources may also be categorized as interpersonal skills, support from seniors, and a psychosocial environment. The Institution’s may also provide resources by providing clarity in job roles, leadership competence and allowing individuals to participate in decision making (Bjaalid et al., 2022). In service sectors, there are two categories of job demands: positive job demands and negative job demands.

This job demand-resource theory’s strain hypothesis has been linked to a wide range of consequences, such as the risk of greater depression and poor quality of sleep (Dutheil et al., 2020), higher burnout, and lower work engagement (Vassos et al., 2019). Researchers also found workplace bullying as an outcome of this strain hypothesis (Balducci et al., 2020; Naseem and Ahmed, 2020). It has been argued that employees try to reduce stress by distancing themselves from stressful situations by violating workplace norms such as withdrawal behavior, social violation or isolation, and putting less effort into work (Notelaers et al., 2013). Such violation of workplace norms arouses others to adopt a negative attitude toward the stressed employee and requires more resources to reduce stress (Notelaers et al., 2013). Such a person may become an easy victim of workplace bullying (Pastorek et al., 2015). Research in Australian, Spanish, and Belgian contexts also related workloads and excessive work demand positively with strain hypothesis (Barlett and Coyne, 2014). Researchers found that these work-related outcomes may arouse stress, which results in exposure to workplace bullying (Baillien et al., 2019).

### Workplace bullying and psychological distress

Research has shown a negative association between workplace bullying and mental health (Lever et al., 2019). Psychological distress often deteriorates mental health, before discussing how workplace bullying and psychological distress are associated, we need to understand what in fact psychological distress is. Psychological distress is a condition full of emotions rendered by high signs of depression and anxiety and frustration. This kind of emotional experiencing is related with work demand constraints which is difficult to meet in routine life (Asaoka et al., 2021; Bano et al., 2021). Work-related bullying is also related with constant criticism, minimum deadlines to meet work demands, extra monitoring of work to make employees realize about their work inefficiencies. This kind of behavior is pretty enough to arouse feelings of irritation, which at later stages leads to emotional exhaustion (Naseem and Ahmed, 2020) and finally into anxiety and frustration.

Not only bullying victims are experiencing emotional disorder and frustration but also the witnesses. Although percentage of bullying victims is high to show psychological disorder (Nielsen and Einarsen, 2012a; Heffernan and Bosetti, 2021). Studies also shown that perpetrators are also developed high symptoms of depression (Wen et al., 2022). Niedhammer et al. (2020) reported that workers who witnessed workplace bullying had three to four times higher depressive symptoms than those who did not personally experience bullying nor witness others being bullied, while victims had eight times higher depressive symptoms than those with no experience or witness of workplace bullying (Harb et al., 2021).

Employees who are working in service sector, particularly in health and education sector; are all the time in touch with patients and students, are experiencing more anxiety and frustrations (Einarsen et al., 2020; Asaoka et al., 2021; Putra and Artini, 2022). Therefore, Employees in service sector are keeping themselves all the time busy to meet challenges of daily changing work demands. Keeping in view the deficiency of information on the mediating role of workplace bullying on psychological distress, through work demand constraints, this study puts an effort to investigate the workplace bullying behavior and its impact on employee’s psychological distress level in service sector employees.

### Big five personality

**FIGURE 1:**
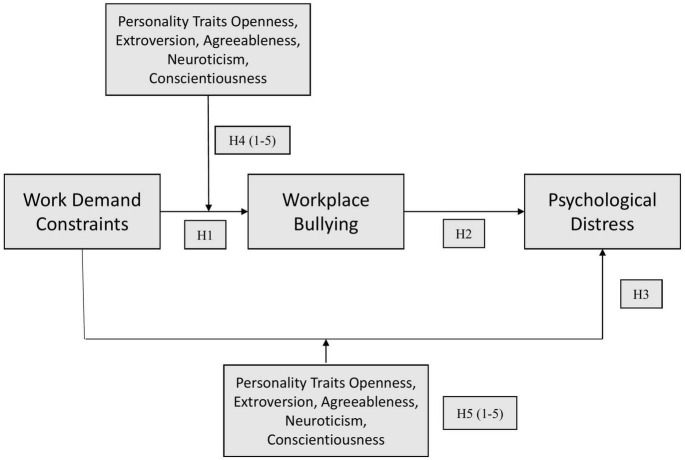
Theoretical framework.

Among the various theories of personality, the Big five model seems to be the most influential model in modern psychology (Ettis and Kefi, 2016). This model shows that personality traits can be designed into the five- broad categories, which include “extraversion (sociable, gregarious, assertive, talkative, active), agreeableness (courteous, trusting, good-natured, cooperative, tolerant), conscientiousness (careful, responsible, organized), neuroticism (anxious, depressed, angry, embarrassed, worried, and insecure), and openness to experience (imaginative, cultured, curious, original, intelligent)” (McCrae and John, 1992; Gómez-Galán and Lázaro-Pérez, 2020). Exploring personality traits of the victims, perpetrators and the witness has been the subject of interest by many research studies. Big five has been linked with both bullying victimization and intimidation (Mitsopoulou and Giovazolias, 2015; van Geel et al., 2017) and aggressive and violent behaviors (Barlett and Anderson, 2012). Agreeableness has been found to be negatively associated with victimization and intimidation (Kokkinos et al., 2016; Koukia, 2020). Emotional instability often expressed by aggressive behaviors both in bullies and victims is linked with intimidation (Hansen et al., 2012) and victimization (Alonso and Romero, 2017). Low scale conscientious leads to bullying victimization (Kokkinos et al., 2016) and perpetration (Koukia, 2020). High level of Extroversion leads to perpetration (Koukia, 2020), While low scale of Extraversion has also been associated with the victimization, (Kokkinos et al., 2016). On the other hand, it has been shown that individuals who experience bullying at workplace leads to reduced agreeableness and increase in neuroticism (Naseem and Ahed, 2021). This shows that workplace bullying and big five personality traits are highly correlated in different forms. However, this study tries to investigate the moderating role of personality traits which itself is an addition in theoretical literature of bullying. The main focus is to know which dimensions of big five personality traits plays a moderating role in the relationship between WDC and workplace bullying. We also checked the moderation of personality traits dimension in the indirect relationship of WDC and psychological distress. Theoretical framework is provided in Figure 1. The hypotheses are:

*H1: WDC is positively linked with workplace bullying.*

*H2: Workplace bullying mediates the linkage between WDC and psychological distress.*

*H3: WDC is positively linked with Psychological distress.*

*H4(1-5): Personality traits (extroversion, agreeableness, openness to experience, neuroticism, and conscientiousness) moderate the positive linkage between WDC and bullying behavior, such that it is less evident for employees with great personality traits.*

*H5 (1-5): Personality traits (extroversion, agreeableness, openness to experience, neuroticism, and conscientiousness) moderate the indirect impact of WDC on psychological* distress *through bullying behavior. Especially, bullying behavior mediates the indirect effects when personality traits are high but not when it is low.*

## Method

### Research approach

This study used a deductive research approach based on survey questionnaire. Deductive approach is used for theory testing or modification (Bryman and Bell, 2015). Questionnaire was based on above hypotheses. This empirical study was designed for descriptive research.

### Questionnaire designing

The objective of this study was to investigate the role of WDC and bullying (direct and indirect) on psychological stress using five dimensions of personality traits as a moderator. A pilot study was conducted to check the reliability and validity of the questionnaire. For the pilot study, we selected fifteen academic professors and fifteen doctors from healthcare and ten forest officers. Their feedback showed the existence of workplace bullying and its negative consequences due to work demand constraints. No specific changes were made in questionnaire. All items that we used in the questionnaire are given in Appendix A. Four Variable measurements includes one independent variables (WDC), one mediating variable (workplace bullying), one moderating variable (Personality Traits), and one dependent variable (psychological distress) were used in this study.

This research used a five-items measure developed by Boyar et al. (2007) to measure work demand constraints. Sample item was “I feel like I have a lot of work demand”. The items used in the study were considered valid because of their alpha value above the standard 0.70. The psychological distress of bullying was measured by using five items from the Beck Anxiety Inventory (Beck et al., 1988). Sample item was, “Feeling constant fear of the worst happening”. Cronbach’s alpha was (0.89). Big Five Personality Traits were measured by a 10-item short version of the Big Five Inventory developed by Rammstedt and John (2007). Sample item was, “I see myself as someone who handles stress well”. Cronbach’s alpha was (0.79). The Negative Acts Questionnaire measured bullying Behaviors (NAQ-R) developed and revised by Einarsen and Raknes (1997). Five items for work related bullying were used for this study. Sample item was, “Persistent criticism of your work and efforts.” The response rate was also measured on five points Likert scale, and its range was never = 1, to weekly = 5. Cronbach’s alpha was (0.87).

### Sample and data collection procedure

In order to collect data, a letter of permission for conducting research was issued from the institution to ensure the confidentiality of the responses. The study’s first author approached the heads of different public and private sector institutions in Lahore, Pakistan to seek permission for collecting data from their full-time, regular employees serving in the respective organizations. The author introduced the purpose of the research and solicited their consent to participate in the research study. To reduce the common method biases identified by Podsakoff et al. (2003), the data was collected by using two wave (15 days interval) time lagged design. By using systematic sampling technique, Participants were invited to fill out the questionnaires about demographics, work demand constraints and workplace bullying at Time 1 (T1). Two weeks later, at Time 2 (T2), participants were asked to complete the Questionnaires for personality traits and psychological distress. The employees provided a self-report response at both times (T1 and T2). At T1, a total of 1,000 questionnaires were distributed. The sample size was calculated using G*POWER software (Faul et al., 2007), and the resulting number is 156 (statistical power = 83%, effect size = 0.02, no of arrows pointing at PD = 5). This shows, any sample size above 156 should have adequate statistical power to draw valid results. Based on G*power formula, we spread 1,000 questionnaires to get maximum responses. Out of those, 920 questionnaires were received, making it a response rate of 92%. Segregating the questionnaires with missing and incomplete data, 870 were identified as usable. Respondents were requested to write their employee no on the questionnaire for matching the data at Time 2. At T2, questionnaires were distributed to those respondents, who responded and completely filled questionnaire in T1 time to get data regarding the remaining study variables. At T2, 870 questionnaires were then distributed to the participants, out of which 810 were received back. 81 questionnaires had missing data so the usable questionnaires were 729 making response rate 60.7%. The respondents consisted of 471 males (61%) and 292 females (39%) with an average age of approximately 31–40 years (42%). It is pertinent to note that the majority of the participants were from private sector (52%) and married (62%). The detail of the demographics in this study is presented in Table 1.

**TABLE 1:** Sample characteristics.

Measure	Items	Frequency	Percentage
Employment sector	Private	379	52%
	Public	349	48%
Gender	Male	471	61%
	Female	292	39%
Job experience	1–5	145	20%
	6–10	306	42%
	11–15	182	25%
	16–20	73	10%
	above	21	3%
Qualification	Higher secondary	106	15%
	Graduation	392	54%
	Masters and MPhil	231	32%
Marital status	Single	275	38%
	Married	454	62%
Age	21–30	334	41%
	31–40	336	42%
	41–50	91	11%
	51–60	49	6%

### Collinearity test

This study handled potential response biasness by conducting data analysis in two-time intervals. The objective was not to irritate the respondents and they can response by proper reading and understanding the questionnaire. This study also addressed the issue of common method bias (CMB), by using a procedural and a statistical remedy as presented by Kock and colleague (Kock and Lynn, 2012; Kock, 2015). They presented a full collinearity test for PLS-SEM as an inclusive procedure whereby, the variance inflation factors (VIFs) of all the model’s latent constructs are generated. An occurrence of a VIF exceeding a 3.3 threshold would indicate pathological collinearity and hence, that the model may be “*contaminated by common method bias*” (Kock, 2015, p. 7). In this study, all VIFs were below the suggested edge, thus suggesting that CMB may not be a threat to the proposed model. Previous empirical research (Anasori et al., 2020) presented an analogous way of assessment of the potential peril of common method bias. The VIF values are given in Table 2.

**TABLE 2:** Factor loadings of variables [per item, Cronbach alpha, composite reliability, and average variance extracted (AVE)].

Constructs	Indicators	Outer loadings	Alpha	Rho A	CR	AVE	VIF’s value
Bullying behavior	BB1	0.811	0.915	0.919	0.937	0.748	2.501
	BB2	0.882					
	BB3	0.911					
	BB4	0.878					
	BB5	0.838					
Conscientiousness	CON1	0.910	0.777	0.779	0.899	0.817	1.532
	CON2						
Extroversion	EXT1	0.915	0.812	0.812	0.914	0.842	1.734
	EXT2	0.920					
Neuroticism	NEU1	0.924	0.830	0.830	0.922	0.855	1.672
	NEU2	0.925					
Openness to experience	OTE1	0.910	0.779	0.780	0.900	0.819	1.451
	OTE2	0.900					
Agreeableness	AGR1	0.853	0.765	0.854	0.891	0.804	2.134
	AGR2	0.938					
Psychological distress	PD1	0.824	0.905	0.907	0.930	0.725	1.862
	PD2	0.849					
	PD3	0.874					
	PD4	0.840					
	PD5	0.871					
Work demand constraints	WDC1	0.802	0.876	0.883	0.909	0.668	1.456
	WDC2	0.835					
	WDC3	0.839					
	WDC4	0.789					
	WDC5	0.820					

1 = agreeableness, 2 = bullying behavior, 3 = consciousness, 4 = extroversion, 5 = neuroticism, 6 = openness to experience, 7 = psychological distress, and 8 = work demand constraints.

## Results

The study applies Smart PLS 3.3.3 for the assessment of measurement and path models and provides model fit indices in terms of R square, Q square, and F square (Hair et al., 2020). The normality of the data was not good so the Smart PLS was used to test structural equation modeling (Richter et al., 2020) because Smart PLS does not require the normality of the data (Sarstedt et al., 2019). The findings of the study are discussed below:

### Assessment of measurement model

Assessment of the measurement model includes both the reliability and validity of the measurement scales. The reliability of the constructs shows the Cronbach alpha and composite reliability of the measurement constructs. Cronbach alpha and composite reliability (international consistency) values should be equal to 0.7 or greater than the threshold value of 0.7 (Sarstedt et al., 2019; Hair et al., 2020). Table 2 shows that the value of each construct in the model was higher than 0.70 (Cronbach alpha) and composite reliability so, we could say that there was good reliability of the measurement constructs. On the other hand, the validity includes both convergent and discriminant validity (Richter et al., 2020). Additionally, convergent validity shows two parameters like one are factor/outer loadings and the second is average variance extracted (AVE), while discriminant validity presents two ways to explain discriminations like one is cross-loadings and the second is HTMT ratio criteria abbreviated as heterotrait monotrait ratio (Sarstedt et al., 2019; Hair et al., 2020). Table 2 presents the results that values of factor/outer loadings were higher than 0.7 on the one hand, and the average variance extracted value of each construct was also higher than 0.5, so we could say that there was good convergent validity. Meanwhile, Table 3 shows that cross-loadings of one construct’ items were higher than the loadings of another construct’ items because the loadings of one construct should be higher than the loadings of another construct in the same column (Hair et al., 2020). Additionally, Table 4 presents that the value of the HTMT (heterotrait-monotrait) ratio should be lower than 0.9, so the value of each construct in the HTMT table was lower than the value of 0.9 in diagonal form (Sarstedt and Cheah, 2019).

**TABLE 3:** Cross loadings.

	1	2	3	4	5	6	7	8
AGR1	0.853	–0.231	0.525	0.594	0.530	0.462	–0.315	–0.162
AGR2	0.938	–0.324	0.686	0.682	0.580	0.523	–0.489	–0.318
BB1	–0.165	0.811	–0.260	–0.137	–0.212	–0.171	0.526	0.486
BB2	–0.334	0.882	–0.395	–0.303	–0.319	–0.314	0.630	0.562
BB3	–0.272	0.911	–0.408	–0.280	–0.367	–0.269	0.587	0.581
BB4	–0.351	0.878	–0.418	–0.294	–0.376	–0.296	0.566	0.549
BB5	–0.237	0.838	–0.443	–0.252	–0.406	–0.325	0.576	0.539
CON1	0.651	0.403	0.910	0.678	0.651	0.568	–0.536	–0.366
CON2	0.590	0.409	0.898	0.552	0.728	0.657	–0.478	–0.395
EXT1	0.636	0.229	0.634	0.915	0.609	0.581	–0.539	–0.287
EXT2	0.677	–0.314	0.618	0.920	0.577	0.467	–0.512	–0.235
NEU1	0.597	–0.369	0.711	0.620	0.924	0.660	–0.486	–0.365
NEU2	0.549	–0.356	0.696	0.576	0.925	0.656	–0.499	–0.405
OTE1	0.508	–0.275	0.645	0.528	0.662	0.910	–0.527	–0.334
OTE2	0.489	–0.307	0.577	0.504	0.625	0.900	–0.474	–0.326
PD1	–0.389	0.705	–0.536	–0.446	–0.560	–0.525	0.824	0.642
PD2	–0.365	0.463	–0.394	–0.458	–0.391	–0.434	0.849	0.616
PD3	–0.396	0.502	–0.523	–0.540	–0.450	–0.510	0.874	0.476
PD4	–0.305	0.647	–0.432	–0.426	–0.397	–0.395	0.840	0.545
PD5	–0.516	0.510	–0.496	–0.569	–0.454	–0.484	0.871	0.598
WDC1	–0.173	0.404	–0.281	–0.103	–0.217	–0.200	0.414	0.802
WDC2	–0.293	0.521	–0.430	–0.354	–0.478	–0.455	0.643	0.835
WDC3	–0.271	0.555	–0.368	–0.250	–0.302	–0.296	0.618	0.839
WDC4	–0.170	0.629	–0.274	–0.155	–0.287	–0.142	0.495	0.789
WDC5	–0.230	0.423	–0.346	–0.263	–0.385	–0.374	0.563	0.820

1 = agreeableness, 2 = bullying behavior, 3 = consciousness, 4 = extroversion, 5 = neuroticism, 6 = openness to experience, 7 = psychological distress, and 8 = work demand constraints.

**TABLE 4:** Heterotrait-monotrait (HTMT) ratio.

	1	2	3	4	5	6	7
**Agreeableness**							
Bullying behavior	0.365						
Consciousness	0.871	0.529					
Extroversion	0.900	0.339	0.857				
Neuroticism	0.774	0.446	0.890	0.788			
Openness to experience	0.708	0.377	0.870	0.718	0.884		
Psychological distress	0.535	0.728	0.666	0.668	0.610	0.656	
Work demand	0.320	0.691	0.504	0.327	0.479	0.434	0.747
Constraints_							

1 = agreeableness, 2 = bullying behavior, 3 = consciousness, 4 = extroversion, 5 = neuroticism, 6 = openness to experience, 7 = psychological distress, and 8 = work demand constraint.

### Assessment of path model direct and mediation analysis

The study applied bootstrapping technique with 1,000 sub-sample and maximum iterations (Sarstedt et al., 2019; Hair et al., 2020). Bootstrapping technique meets three criteria like regression value (r) should be between + 1 and −1, t-value should be higher than + 1.96 in case of 0.5 significance level and 95% confidence interval, and *p*-value should be lower than 0.05, means *p* < 5% (Sarstedt et al., 2019; Hair et al., 2020; Richter et al., 2020). Smart PLS provides specific indirect effects (Richter et al., 2020). Therefore, the study consults the direct effects in case of a direct link between exogenous construct and indigenous construct; however, it consults special, indirect effects in case of mediating the relationship between exogenous construct and endogenous construct. Table 5 present that work demand constraints significantly and positively influenced bullying behavior (b = 0.535, t-value = 12.874, *p*-value = 0.000) in turn, significantly and positively influenced psychological distress (b = 0.355, t-value = 9.006, *p*-value = 0.000). Additionally, work demand constraints significantly and positively and directly influenced psychological distress (b = 0.358, t-value = 9.716, *p*-value = 0.000). Furthermore, bullying behavior was found to have a significant and positive mediating role between work demand constraints and psychological distress (b = 0.190, t-value = 6.244, *p*-value = 0.000). Therefore, it was proved that work demand constraints affected bullying behavior, and it was the highest side effect of work demand constraints on bullying behavior. Meanwhile, work demand constraints also affect psychological distress, and it means the work demand constraints create psychological distress in employee’ lives. Third, bullying behavior was the third severe factor creating psychological distress in employees’ lives.

**TABLE 5:** Regression coefficients.

	Original sample (O)	Standard deviation (STDEV)	T statistics (|O/STDEV|)	*P*-values
Work demand constraints_ -> Bullying behavior	0.535	0.042	12.874	**0.000**
Bullying behavior -> Psychological distress	0.355	0.039	9.006	**0.000**
Work demand constraints_ -> Psychological distress	0.358	0.037	9.716	**0.000**
Work demand constraints_ -> Bullying behavior -> Psychological distress	0.190	0.030	6.244	**0.000**

*p* < 0.05.

### Moderation analysis

By analyzing the moderating relationships, Table 6 presents that extroversion did not significantly moderate the link between work demand constraints and bullying behavior (b = 0.066, t-value = 1.105, *p*-value = 0.270). Second, agreeableness was not found to have a moderating role between work demand constraints and bullying behavior (b = 0.061, t-value = 1.282, *p*-value = 0.200). Third, Consciousness was not found to have a significant moderated role between work demand constraints and bullying behavior (b = −0.002, t-value = 0.028, *p*-value = 0.977). Forth, neuroticism did not significantly moderate the link between work demand constraints and bullying behavior (b = −0.041, t-value = 0.746, *p*-value = 0.456). Fifth, openness to experience was found to have a significant and negative moderated link between work demand constraints and bullying behavior (b = −0.114, t-value = 2.495, *p*-value = 0.013).

**TABLE 6:** Regression coefficients for moderation.

	Original sample (O)	Standard deviation (STDEV)	T statistics (|O/STDEV|)	Coefficients of moderation	*P*-values
Work demand constraints*Extroversion - > Bullying behavior	0.066	0.060	1.105	**0.31**	**0.270**
Work demand constraints*Agreeableness - > Bullying behavior	0.061	0.048	1.282	**0.55**	**0.200**
Work demand constraints*Consciousness - > Bullying behavior	−0.002	0.076	0.028	**0.37**	**0.977**
Work demand constraints*Neuroticism - > Bullying behavior	−0.041	0.055	0.746	**0.41**	**0.456**
Work demand constraints*Openness to experience - > Bullying behavior	−0.114	0.046	2.495	**0.11**	**0.013**
Work demand constraints*Extroversion - > Psychological Distress	−0.008	0.035	0.224	**0.58**	**0.823**
Work demand constraints*Agreeableness - > Psychological distress	−0.154	0.037	4.222	**0.15**	**0.000**
Work demand constraints*Consciousness - > Psychological distress	0.063	0.046	1.375	**0.29**	**0.170**
Work demand constraints*Neuroticism - > Psychological distress	−0.059	0.034	1.725	**0.35**	**0.085**
Work demand constraints*Openness to experience - > Psychological distress	0.096	0.032	3.021	**0.09**	**0.003**

*p* < 0.05.

By analyzing the moderating role between work demand constraints and psychological distress, the study shows that extroversion did not significantly moderate between work demand constraints and psychological distress (b = −0.008, t-value = 0.224, *p*-value = 0.823). As well, agreeableness was found to have a significant and negative moderating relationship between work demand constraints and psychological distress (b = −0.154, t-value = 4.222, *p*-value = 0.000). Additionally, Consciousness (b = 0.063, t-value = 1.375, *p*-value = 0.170), and neuroticism (b = −0.059, t-value = 1.725, *p*-value = 0.085) were not found to have a significant and negative moderating relationship between work demand constraints and psychological distress. Most interestingly, openness to experience was also found to have a significant moderating role between work demand constraints and psychological distress (b = 0.096, t-value = 3.021, *p*-value = 0.003). Finally, it was found that openness to experience was one of the factors that negatively affected bullying behavior through work demand constraints. In the second stage, agreeableness and openness to experience were two of the five factors that showed moderation. In which agreeableness negatively and significantly moderates the link between work demand constraints and psychological distress. While, openness to experience positively and significantly moderates the link between work demand constraints and psychological distress.

## Discussion

This study aims to increase human sustainability in organizations to contribute to social dimensions of sustainability by reducing bullying behavior at the workplace. Human sustainability focuses on employee well-being. We have discussed the factor, bullying behavior and its antecedent WDC, which negatively impacts human sustainability. By avoiding this factor or reducing the intensity of this variable, workplace bullying, we can increase human development and hence play a role in human sustainability (Eizenberg and Jabareen, 2017).

This research investigates the negative role of workplace bullying in sustainable human development in the service sector, particularly employees’ exposure to workplace bullying according to the work environment hypothesis. Based on mediated moderation analysis of the service sector’s employees; in different departments, this research found that work demand constraints, personality traits, and psychological distress are significant correlations to workplace bullying. Results show a high percentage of bullying victimization in the service sector.

This study proposed a theoretical framework for the work demand constraints model (WDC) that examined the linkage between four significant variables, i.e., work demand constraints, bullying behavior, psychological distress, and personality traits. This study outcome shows work demand constraints are a great risk factor for involving in bullying behaviors. This is also supported by existing research (Budin et al., 2013; Spagnoli and Balducci, 2017). The service sector environment can be portrayed as fast-paced and highly stressed than others, and more performance-oriented as employees directly interact with customers. Work demand constraints in the form of workload, excessive work stress, Pressure to meet deadlines/targets, and fight for survival may deteriorate the work environment, which may act as the antecedent of workplace bullying. Previous research has found that role conflict, low job control, and job strains (Balducci et al., 2020) are significantly related to workplace bullying.

A significant contribution of this study is identifying the underlying mechanism in the association of work demand constraints -workplace bullying through personality traits. The study proved that only openness to experiences in personality traits is the primary resource that plays a moderator role in the relation between work demand constraints and workplace bullying. Meanwhile, agreeableness and openness to experiences were moderating the relationship between work demand constraints and psychological distress. Identifying personality traits (agreeableness and openness to experiences) as a psychological motive between these associations as well as discussing their essential role in predicting employee’s becoming a victim of workplace bullying in service sector is a major contribution of this study. Which may provide guidance to top management and employers in policymaking to identify personality traits during interviews. Employers and policy makers may also develop personalities by providing training on personality development. This shows that human sustainability can be increased by developing personality traits in the workplace.

Our study results supported Pabón-Carrasco et al. (2020) and Mitsopoulou and Giovazolias’s (2015) findings that personality traits (openness to experiences) have a linkage with bullying behavior. While Openness to experiences and agreeableness have association with psychological distress which supports the thought of (Naseem and Ahed, 2021). However, our results were not supporting the thoughts of (Kokkinos et al., 2016; Koukia, 2020), as extroversion and conscientious were insignificant. However, our study is of its kind, which identifies the moderating effect of personality traits on the WDC and bullying relationship. This shows that employees with strong personality traits minimize the effect of work demand constraints on psychological distress and show less psychological irritating outcomes. These results suggested that improving personality traits (agreeableness and openness to experiences) among employees of service sectors helps to avoid from becoming bullying victims. This also shows that personality traits (agreeableness and openness to experiences) play a significant role in human sustainability in organizations.

The results show that work demand constraints are positively related to workplace bullying, and supported (Heffernan and Bosetti (2021), Niedhammer et al., 2020) and Samsudin et al. (2020) school of thought that bullying is the characteristic organizational hypothesis. Our study also supports Bashir and Hanif’s (2019) findings which found bullying was related to psychological strain. Our results were contrary to the finding of (Nielsen and Einarsen, 2018), who found that concerning health and other psychological distress, individual dispositions and organizational characteristics play a buffering role.

### Theoretical implications

Research outcomes entail a significant effect on psychological distress (PS) through workplace bullying (WB). Embedded with the proposition of job demand theory, this study provides a theoretical contribution in the context of the WDC-WB relationship and subsequently, WB-PS linkage. These outcomes not only support the theoretical grounding of this study but also further provides a deep understanding of the effects related to psychological distress. Furthermore, this study develops the theoretical understanding of the moderation effects that are analyzed in this study model. Therefore, this study contributes to both organizational psychology and service sector organizations in terms of literature and theoretical development and helps in human sustainability by improving mental health. When employees possess certain work demand constraints, they may combat or fall victim to bullying but have strong personality traits they may avoid becoming a bullying victim. Similarly, employees with strong personality traits (agreeableness and neuroticism) may have fewer psychological impacts than fewer personality traits. Our results stress that a strong personality trait (agreeableness and neuroticism) is a buffer in bullying. Our results are in-line with the thought of prior studies (Baillien et al., 2019; Samsudin et al., 2020) and support the organizational factors of workplace bullying. Our findings suggest that how personality traits help individuals at workplace to reduce the bullying effects and renders converting them into psychological distress.

### Practical implications

Our study suggests few practical implications for policymakers, executive officers, employers, and authorities. Firstly, remodeling the educational curriculum is required to eradicate bullying from the grassroots level; ethics, morality, and social values should be incorporated into the syllabus of business studies. Secondly, periodically personality development training should be provided to all employees serving at all levels, especially senior employees, which will help them survive in this growing and diverse environment.

Thirdly, employers should introduce a reporting system where employees may report a complaint against bullying behavior for organizations’ sustainability. They must have surety organizations will provide shelter from emotional halt caused by bullying behavior by making the environment safe. Salin (2015) has also focused on redesigning the work environment and has deliberated that these personality development programs and training will help change the workplace environment. Fourthly, providing psychological counseling services at the organizational level or having an agreement with occupational health care to help employees in prevention against bullying may be a good remedy and increase human sustainability in organizations.

### Limitations and future directions

Since cross-sectional research has limitations concerning understanding prediction and criterion linkage among variables. Methodological constraints of the current study open new pathways for future studies such as: First longitudinal designs may be an appropriate approach in the future (Nielsen and Einarsen, 2012b). In addition to longitudinal design, short and long intervals might have better potential concerning time laps to prosper relationships among bullying and its consequences. Also, workplace bullying is a continuous process that needs a longer period. Diary studies with repeated measurement points may also provide a valuable approach. Besides, most studies use a self-reported questionnaire with a subjective approach; future research requires an objective approach.

Furthermore, personality traits (Extroversion, consciousness, and neuroticism) were not found to have a moderating role among work demand constraints, bullying behavior, and psychological distress; however, future studies may use some other variables such as emotional intelligence (Bunnett, 2021), leadership skills (Fontes et al., 2019), and deep surface acting behaviors (Khurram et al., 2020) as a buffer to reduce bullying effects.

This study was conducted in service sector, where employees need to direct interact with customers. In manufacturing sector, job demands and resources may be used in a different perspective. The current study used a job demand theory, future studies may use a combination of job demand and job resource theory or job demand support model. Organizational support system may be helpful for employees to make them less psychologically sick.

### Conclusion

Our study significantly contributes to the literature on organizational psychology and human sustainability by discussing the importance of workplace bullying. Individuals with strong personality traits (agreeableness and openness to experiences) may be beneficial in either avoiding being a bullying victim or its consequences such as psychological distress. Our study has theoretical and practical implications for employees and employers in organizations. Our empirical results demonstrate that work demand constraints are positively linked with bullying behavior and psychological distress. However, these two strong personality traits play a buffer role in this relationship. Excessive research stressed workplace bullying distress such as physical exhaustion, fatigue, mental illness, sleep-related problems, and disorders, but our finding recommends that personality traits play a role in controlling the psychological effect of bullying.

## Data availability statement

The raw data supporting the conclusions of this article will be made available by the authors, without undue reservation.

## Ethics statement

This study was reviewed and approved by the University Research Committee. The patients/participants provided their written informed consent to participate in this study.

## Author contributions

KN drafted the idea, collected the data, and analyzed the results. MA handled all the revision asked by reviewers and documented the manuscript for grammatical and language issues. Both authors contributed to the article and approved the submitted version.

## Appendix: Survey questionnaire

### Survey part 1

#### Demographics

1. Employment Sector. public/private.
2. Age. 21–30, 31–40, 41–50, 51–60.
3. Gender. Male/Female.
4. Job Experience. 1–5, 6–10, 11–15, 16–20, above.
5. Qualification. Higher secondary/Graduation/Masters and MPhil.
6. Marital Status. Single/Married.

“Bullying takes place when one or more persons systematically and over time feel that they have been subjected to negative treatment on the part of one or more persons, in a situation in which the person(s) exposed to the treatment have difficulty in defending themselves against them”. It is not bullying when two equally strong opponents are in conflict with each other.

Over the last six months, how often have you or any other employee been subjected to the following negative acts at work? Please circle the number(s) that best corresponds with your experience over the last six months.

**TABLE A1:**

S#	Work related bullying at workplace	Never	Now and then	Monthly	Weekly	Daily
1	Having your opinions ignored.	1	2	3	4	5
2	Persistent criticism of your work and efforts.	1	2	3	4	5
3	Being given tasks with unreasonable deadline.	1	2	3	4	5
4	Being exposed to unmanageable workload.	1	2	3	4	5
5	Being shouted at or target of spontaneous anger.	1	2	3	4	5

**TABLE A2:**

S#	Work demand constraints items	Strongly disagree	disagree	Neutral	Agree	Strongly agree
1	My job requires all of my attention.	1	2	3	4	5
2	I feel like I have a lot of work demands.	1	2	3	4	5
3	My work requires Excessive workloads and multiple skill sets.	1	2	3	4	5
4	I am given a lot of work to do.	1	2	3	4	5
5	I feel like I have a Pressure to meet deadlines/targets.	1	2	3	4	5

### Survey part 2

**TABLE A1:**

S#	Personality traits I see myself as someone who…	Strongly disagree	disagree	Neutral	Agree	Strongly agree
1	… is reserved	1	2	3	4	5
2	… is generally trusting	1	2	3	4	5
3	… tends to be lazy	1	2	3	4	5
4	… is relaxed, handles stress well	1	2	3	4	5
5	… has few artistic interests	1	2	3	4	5
6	… is outgoing, sociable	1	2	3	4	5
7	… tends to find fault with others	1	2	3	4	5
8	… does a thorough job	1	2	3	4	5
9	… gets nervous easily	1	2	3	4	5
10	… has an active imagination	1	2	3	4	5

**TABLE A2:**

S#	Psychological distress I see myself as someone who often feels…	Strongly disagree	disagree	Neutral	Agree	Strongly agree
1	Wobbliness in legs Unable to relax	1	2	3	4	5
2	Numbness or tingling Feeling hot	1	2	3	4	5
3	Fear of the worst happening	1	2	3	4	5
4	Difficulty in breathing and Fear of dying	1	2	3	4	5
5	Fear of losing control	1	2	3	4	5
